# Importance of basic life support training for first and second year medical students–a personal statement–

**Published:** 2010-11-25

**Authors:** RO Tipa, G Bobîrnac

**Affiliations:** ‘Carol Davila’ University of Medicine and Pharmacy, Bucharest Romania

**Keywords:** basic life support, education, e–learning, student curricula, medical students, social support

## Abstract

**Introduction:** Current studies show that there is a significant lack of knowledge regarding the typical signs and risk factors associated with serious medical conditions among medical students and laypersons. Basic life support consists of a number of medical procedures provided to patients with life threatening conditions of the body that cause pain or dysfunction to the person.

**Further developments:** In spite of the fact that this programme is not included in the curricula, students might receive this information from various persons, even though these persons are not specialized in the domain of giving first aid. Learning medicine without placing patients at an increased risk of complications is of utmost importance in the medical profession. High–fidelity patient simulators can potentially achieve this, and, therefore they are increasingly used in the training of medical students. Recent studies regarding simulation training show that the simulation–based intervention offers a positively evaluated possibility to enhance students' skills in recognizing and handling emergencies improving the ability to manage medical emergencies.

**Conclusion:** Understanding BLS courses and more than that, practicing these techniques is by far the most challenging task confronting first aid.

Taking everything into consideration, we believe that an adequate education in first aid and basic life support should be considered an essential aspect of the medical curriculum.

## Introduction

Current studies show that there is a significant lack of knowledge regarding the typical signs and risk factors associated with serious medical conditions among medical students and laypersons. [1] There is an urgent need to establish learning objectives in order to encourage students to complete BLS courses during their education. 

In the case of an emergency, fast and structured patient management is crucial for a patient's outcome. [2] In order to manage common emergencies every medical student and graduate should possess basic knowledge of emergency care and the skills necessary for dealing with these situations. Adequate education in first aid and basic life support (BLS) should be considered as an essential aspect of the medical curriculum. 

Furthermore, the most important BLS skill, good quality chest compressions, was retained by significantly more students who were taught the new resuscitation guidelines according to the new curriculum [3], although no such studies can be performed in the Romanian medical teaching system, since no BLS formal curricula has been implemented for lower or higher year medical students. A comfortable solution for this might be the E–learning [4] proven to improve both the knowledge and the competence of medical students in pediatric cardiopulmonary resuscitation, at least in the simulation environment. 

Although this might be a low cost and easy implementation solution, recent medical school graduates report lack of self–confidence in their ability to perform common procedures upon entering residency training. Implementation of a medical school procedure course to increase exposure to procedures may address this challenge; simulation exercises for second–year medical students may be a valuable tool to increase knowledge and student self–confidence at a key transition period prior to beginning clerkship experiences.[5] Further research is needed to prove long–term educational benefits of simulation interventions in the preclinical setting. [6] 

Basic life support consists of a number of medical procedures provided to patients with life threatening conditions of the body, that cause pain or dysfunction to the person. All these techniques are focused on helping patients or sustain life until more precise medical treatment can begin. 

Even though BLS does not include extensive medical supervision or treatment, including the use of invasive procedures or drugs, only those who attended a BLS training program can provide it. Their certification allows them to provide basic life–saving and life–sustaining interventions until the trained medical personnel gives them the full medical care known as advanced life support (ALS). 

Usually, these medical techniques, which refer to the initial assessment, airway maintenance and ventilation, breathing, circulation and chest compression, consist of a few steps that must be followed. And these steps can easily be taught in a 12 to 24 h class. (Table 1)

**Table 1:** Basic life support techniques and mnemonics as set by the 2009 American Heart Association Protocols

Mnemonic	Approach
D	Secure the **DANGER** area. Ensure the safety of the victim, the rescuer, and any bystanders
R	Check the victim for a **RESPONSE** by gently shaking the victim's shoulders and asking loudly ‘Are you all right?’ If the victim responds, leave him in the position in which he was found provided there is no further danger, try to find out what is wrong with him and get help if needed, and reassess him regularly.
A	If the victim does not respond, turn him on to his back and open the **AIRWAY** using the head tilt and chin lift. Shout for help.
B	Look, listen and feel for normal breathing for no more than 10 seconds. If the victim is **BREATHING** normally, turn him into the recovery position and get help. Continue to check for breathing
C	If the victim is not breathing normally, call for an ambulance, then give 30 chest **COMPRESSIONS** at a rate of about 100 per minute. After 30 chest compressions, give 2 rescue breaths, and continue to alternate between 30 chest compressions and 2 breaths.
D	Continue resuscitation until qualified help arrives with a **DEFIBRILLATOR**, the victim starts breathing normally, or you become exhausted.

All these techniques can be provided without any medical equipments but they typically include considerations for patient's transport such as various forms of immobilization to prevent additional injury, including cervical collars, splinting limbs, and full body splints.

BLS protocols continue until (1) the patient regains a pulse, (2) the rescuer is relieved by another rescuer of equivalent or higher training, (3) the rescuer is physically too tired to continue CPR, or (4) the patient is pronounced dead by a medical doctor [7].

## Further developments

There is an important number of people who imply people suffering from a lack of an appropriate care in an uncontrolled environment emergency. To begin with, the main important advantage of receiving a BLS training, this class is useful for everyone, not only for personal lives but also for others.  More than that, the course has proven to be beneficial to a wide range of persons including parents who do not want to be let down by forgotten or weak resuscitation skills.

In spite of the fact that this programme is not included in the curricula, students might receive this information from various persons, even though these persons are not specialized in the domain of giving first aid. Therefore, some of the attendants might not acquire the proper/accurate information, and, this may lead to medical errors and a low self–confidence because of the low level of knowledge acquired.  On the other hand, there are associations, which organize these kinds of programs not only for students but also for the medical personnel or for anyone eager to learn about first aid.  We are in the category of the persons who received a BLS training in an organized way; that is why we strongly recommend that BLS should be included in the curricula. 

Learning medicine without placing patients at an increased risk of complications is of utmost importance in the medical profession. High–fidelity patient simulators can possibly achieve this, and are therefore used very often in the training of medical students. Preclinical medical students have a minimal exposure to clinical rotations and commonly feel anxious and apprehensive when starting their clinical years. [8] Implementation of a medical school procedure class to increase the exposure to procedures may address this challenge [9], and also create a more properly organized and secure learning environment in higher years of medical school, because of the initiation of students in the art of patient handling. We strongly suggest the need for implementation of classes that develop specific patient communication and approach skills along with the theory of history taking and basic life support, both for assuring the fresh clinical year student the self–confidence necessary in a hospital ward and for assuring a more secure clinical university environment.

Recent studies regarding simulation training show that the simulation–based intervention offers a positively evaluated possibility to enhance students' skills in recognizing and handling emergencies improving the ability to manage medical emergencies. [9] And, we must recognize the fact that, in the case of acute medical emergencies, seconds count, and these may be the seconds needed for a doctor to arrive next to the patient that a student is examining or taking a medical history on. 

## Conclusions

Understanding BLS courses and, more than that, practicing these techniques is by far the most challenging task confronting firs aid. Either by theoretical classes, practical training or virtual e–education, we feel that basic life support techniques must be acquired as soon as possible in medical school, because their core meaning is that of saving a life, the main reason why all students chose the medical profession

Taking everything into consideration we believe that the adequate education in first aid and basic life support should be considered as an essential aspect of the medical universities' curriculum. Being students, we feel that this might improve both the state–of–mind aspects of the medical students, their eagerness to learn, their passion for the medical art and also their clinical skills. As stated, implementing such a strategy might be also useful during clinical stages, as all parts implicated in the clinical act should be trained to handle basic life support emergencies, even for a second, since that second has been very well proven its importance.

## References

[1] Businger A (2010). Students' knowledge of symptoms and risk factors of potential life–threatening medical conditions. Swiss Med Wkly.

[2] Ruesseler M, Weinlich M (2010). Simulation training improves ability to manage medical emergencies. Emerg Med J.

[3] Papaioannou A (2010). Basic life support skill retention by medical students: a comparison of two teaching curricula. Emerg Med J.

[4] O'Leary FM, Janson P (2010). Can e–learning improve medical students' knowledge and competence in paediatric cardiopulmonary resuscitation? A prospective before and after study. Emerg Med Australas.

[5] Promes SB, Chudgar SM, Grochowski CO (2009). Gaps in procedural experience and competency in medical school graduates. Acad Emerg Med..

[6] Halm BM, Lee MT, Franke AA (2010). Improving medical student toxicology knowledge and self–confidence using mannequin simulation. Hawaii Med J..

[7] Tintinalli JE, Kelen GD, Stapczynski JS (2004). Emergency Medicine: A Comprehensive Study Guide.

[8] Promes SB, Chudgar SM, Grochowski CO (2009). Gaps in procedural experience and competency in medical school graduates. Acad Emerg Med..

[9] Ruesseler M, Weinlich M (2010). Simulation training improves ability to manage medical emergencies. Emerg Med J..

